# Evaluation of Propagation Characteristics Using the Human Body as an Antenna

**DOI:** 10.3390/s17122878

**Published:** 2017-12-11

**Authors:** Jingzhen Li, Zedong Nie, Yuhang Liu, Lei Wang, Yang Hao

**Affiliations:** 1 Shenzhen Institutes of Advanced Technology, Chinese Academy of Sciences, Shenzhen 518055, China; lijz@siat.ac.cn (J.L.); yh.liu2@siat.ac.cn (Y.L.); wang.lei@siat.ac.cn (L.W.); 2 School of Electronic Engineering and Computer Science, Queen Mary College, University of London, London E1 4NS, UK; y.hao@qmul.ac.uk

**Keywords:** propagation characteristics, human body, antenna, inhomogeneous model, simulation and measurement

## Abstract

In this paper, an inhomogeneous human body model was presented to investigate the propagation characteristics when the human body was used as an antenna to achieve signal transmission. Specifically, the channel gain of four scenarios, namely, (1) both TX electrode and RX electrode were placed in the air, (2) TX electrode was attached on the human body, and RX electrode was placed in the air, (3) TX electrode was placed in the air, and RX electrode was attached on the human body, (4) both the TX electrode and RX electrode were attached on the human body, were studied through numerical simulation in the frequency range 1 MHz to 90 MHz. Furthermore, the comparisons of input efficiency, accepted efficiency, total efficiency, absorption power of human body, and electric field distribution of different distances of four aforementioned scenarios were explored when the frequency was at 44 MHz. In addition, the influences of different human tissues, electrode position, and the distance between electrode and human body on the propagation characteristics were investigated respectively at 44 MHz. The results showed that the channel gain of Scenario 4 was the maximum when the frequency was from 1 MHz to 90 MHz. The propagation characteristics were almost independent of electrode position when the human body was using as an antenna. However, as the distance between TX electrode and human body increased, the channel gain decreased rapidly. The simulations were verified by experimental measurements. The results showed that the simulations were in agreement with the measurements.

## 1. Introduction

With the development of radio frequency (RF) technology and body sensor network (BSN), the interaction of RF electromagnetic field with the human body has been an active research topic for many years [1,2]. Parts of these studies are focused on the electromagnetic power absorption by the whole body or parts of the body [3,4,5,6,7]. These studies have provided a comprehensive analysis between the power absorption and human body, which are helpful to understand the effects of RF radiation on the human body.

On the other hand, considering the fact that the human tissues are lossy media [8,9,10], the electric current and electric field are induced in the human body when the human body is exposed to the RF electromagnetic field [11,12]. Therefore, besides the electromagnetic power absorption in human body, the human body may be used itself as an antenna or medium for signal transmission. In recent years, some researchers have demonstrated the propagation characteristics using the human body itself as the transmission medium, which is referred to as human body communication (HBC) or intra-body communication (IBC) [13,14,15,16,17]. However, in the above studies, the transmitter and receiver of HBC are acquired to be located on the same human body due to the fact that the electromagnetic field the human body in HBC technology. In other words, it is indicated that the above studies are mainly suitable for the signal transmission in the same individual rather than among different individuals. Thus far, few researchers have tried to characterize the human body as an antenna to achieve the signal transmission among different individuals. In [18], the authors demonstrated the feasibility of energy harvesting from ambient electromagnetic wave using the human body as a receiving antenna. However, only the power spectral density was acquired in the paper, and therefore the propagation characteristics were still not clear when the human body was used as an antenna. In [19], the human-body equivalent monopole antenna, which was made by different combinations of the rectangular blocks, was proposed as a human surrogate. Furthermore, the induced ankle current of model was measured in the frequency range 30 MHz to 100 MHz when the model was exposed to the electromagnetic field. In [20], the human body was demonstrated as an equivalent cylindrical monopole antenna which was grounded on a highly conductive ground plane. The reflection coefficients of antenna were investigated through the finite-difference time-domain (FDTD) algorithm when the frequency was from 10 MHz to 110 MHz. In addition, the human body was used as an antenna to achieve the wireless implant communication among different individuals in [21]. Specifically, a toroidal inductor at the cross section of the ankle was used to excite electromotive force inside the human body when the frequency was from 1 MHz to 70 MHz. In addition, the receiving toroidal inductor was at the ankle of the other individual. As previously mentioned [19,20,21], it is indicated that the human body can be represented by a monopole antenna to study the propagation characteristics. However, it requires that the human body stands on a metallic plane or ground plane. Meanwhile, the transmitter and receiver should be fixed on the bottom of the human body (i.e., ankle or sole) which corresponds to the feed point of monopole antenna when the human body is modeled as a monopole antenna. Once the position of transmitter or receiver is changed, the human-body equivalent model of monopole antenna may be unavailable. Furthermore, thus far, the factors affecting propagation characteristics are still ambiguous when the human body is used as an antenna. Therefore, in order to better understand the characteristics of human body when it is utilized as an antenna and provides a prospective communication solution for BSN, a comprehensive investigation is necessary and important.

In this paper, we mainly focused on the propagation characteristics when the human body was employed as an antenna to achieve the signal transmission among different individuals. Specifically, different from the previous investigations which the human body is regarded as a monopole antenna and the power is injected into the human ankle or sole by using current directly, the power is coupled into the human body through electrode in this paper, and then the power is radiated out of the body when the human body is utilized as a transmitting antenna. Similarly, the power is coupled into the receiver when the human body is used as a receiving antenna. The advantage of this method is that the positions of the transmitter and the receiver are alterable, so they are no longer limited to the human ankle or sole. The contribution and originality of this paper was summarized as follows. Firstly, the propagation characteristics were investigated when the human body was utilized as an antenna in the frequency range from 1 MHz to 90 MHz. Secondly, the factors affecting propagation characteristics, including the different human tissues, the electrode position on the human body, and the distance between electrode and human body were studied in this paper.

The remainder of this paper is organized as follows. In Section 2, we will present the inhomogeneous human body model and the simulation setup under different conditions. In Section 3, the experimental measurement will be reported. Section 4 discusses and gives a detailed analysis of both simulation and measurement results. Finally, the conclusions are drawn in Section 5.

## 2. Simulation Setup

### 2.1. Human Body Model Proposed

**Figure 1:**
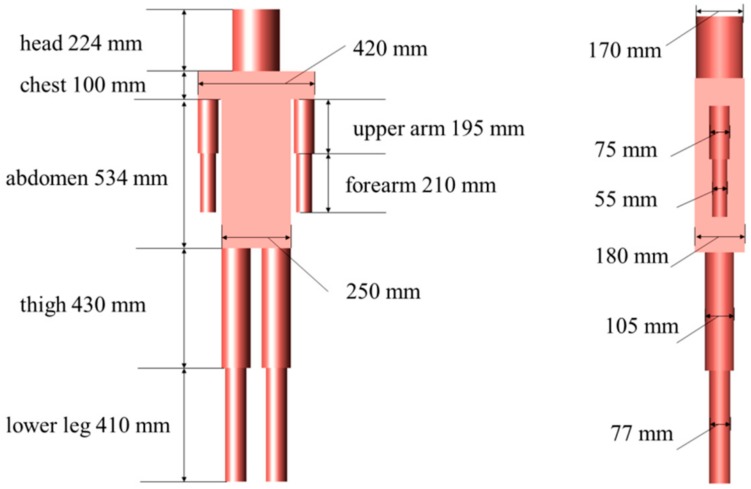
The dimension and body parts of human body model.

In this work, an inhomogeneous human body model, which consists of head, chest, abdomen, upper arm, forearm, thigh, and lower leg, was proposed to explore the propagation characteristics when the human body was used as an antenna. As shown in Figure 1, the height of the human body model was 1698 mm, which corresponded with the average height of an adult [22].The tissue layers and thicknesses of different body parts in this model are listed in Table 1 [23].

### 2.2. Simulation Setup for Four Scenarios

**Figure 2:**
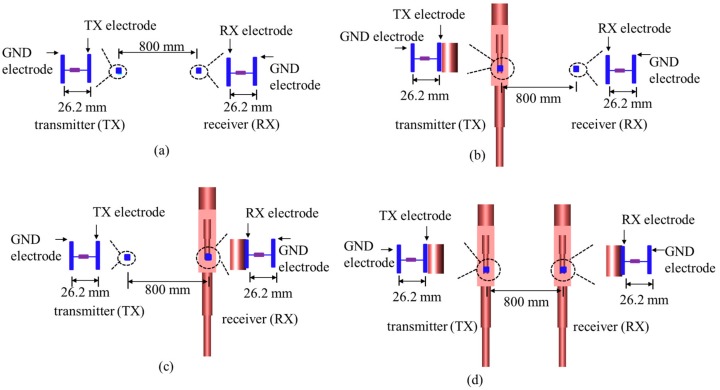
The simulation setup of four scenarios: (**a**) Scenario 1: TX electrode without human body, RX electrode without human body; (**b**) Scenario 2: TX electrode with human body, RX electrode without human body; (**c**) Scenario 3: TX electrode without human body, RX electrode with human body; (**d**) Scenario 4: TX electrode with human body, RX electrode with human body.

In order to better evaluate the propagation characteristics when the human body was utilized as an antenna, as demonstrated in Figure 2, four scenarios were considered in this section. Namely, in Scenario 1, both the transmitter (TX electrode and GND electrode) and receiver (RX electrode and GND electrode) were exposed to the air. In Scenario 2, the TX electrode of the transmitter was attached to the human body, and GND electrode was floated, and then the human body was regarded as a transmitting antenna to transmit signal. Additionally, the signal was received by the RX electrode directly which was exposed to the air. The setup of Scenario 3 was in opposition to that of Scenario 2. In Scenario 4, the TX electrode and RX electrode were attached on a different human body, and the GND electrodes were suspended in the air. The numerical simulations of four aforementioned scenarios were performed by using electromagnetic software XFDTD which is based on FDTD method. The size of TX electrode, RX electrode, and GND electrode in the simulation was 34 mm × 34 mm × 0.2 mm. The distance between TX electrode and GND electrode was 26.2 mm. Similarly, the distance between RX electrode and GND electrode was also 26.2 mm. The TX electrode was excited by a voltage source, and the waveform of voltage source was a broadband signal. Meanwhile, there was a resistance load between the RX electrode and GND electrode. The distance between the transmitter and receiver in all four scenarios was 800 mm. The channel gain between the transmitter and receiver was investigated when the frequency was from 1 MHz to 90 MHz. Considering that the resonant frequency of the human body was mainly between 40 MHz and 60 MHz [12,20], the frequency which was from 1 MHz to 90 MHz was selected as the research object in this paper because it could include the resonant frequency of the human body.

### 2.3. Simulation Setup for the Influence of Electrode Position

**Figure 3:**
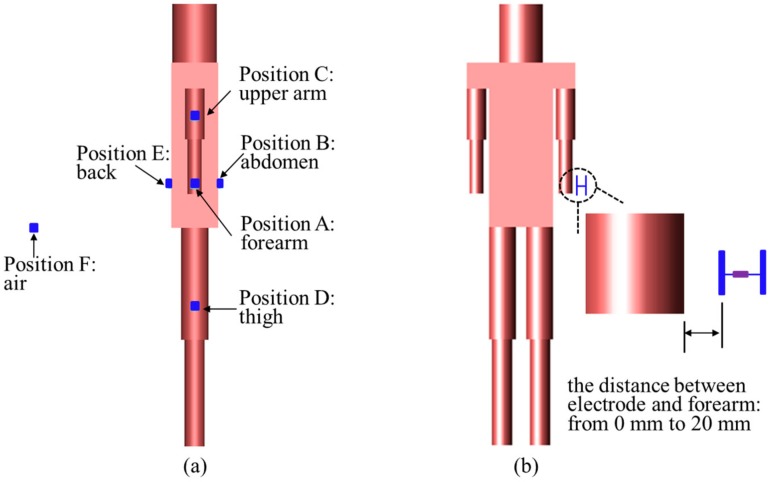
(**a**) Simulation setup about the positions of electrode; (**b**) simulation setup about the distance between electrode and human body.

In this section, we mainly concentrated on the influence of electrode position when the human body was employed as an antenna. As will be shown later, the propagation characteristics of Scenario 2 and Scenario 3 are quite similar. Therefore, to save the computation, the influence of electrode position under Scenario 3 and Scenario 4 were studied respectively in this section. Figure 3a shows the positions of electrode. Specifically, For Scenario 3, the TX electrode and GND electrode were placed in the air (F), and the RX electrode was attached on the different parts of the human body, which were forearm (A), abdomen (B), upper arm (C), thigh (D), and back (E). Similarly, for Scenario 4, the positions of TX electrode and RX electrode were located on the different parts of the human body. The TX electrode was excited by a voltage source. The waveform of voltage source was a sinusoid, of which the frequency was 44 MHz. There was a resistance load between the RX electrode and the GND electrode. The convergence of calculation was defined as −30 dB in FDTD simulation.

### 2.4. Simulation Setup for the Influence of Distance between Electrode and Human Body

As illustrated in Figure 3b, the influence of the distance between the electrode and the human body was studied in this section. Details of simulation setup are as follows. For Scenario 3, the TX electrode were placed in the air and fixed on the specified position, and the RX electrode was placed on the surface of the human forearm. Furthermore, the distance between the RX electrode and the forearm was variable, which was from 0 mm to 20 mm. The TX electrode was fed by a voltage source of which the waveform is a 44 MHz sinusoid. In addition, the simulation setup of Scenario 4 was similar to Scenario 3.

## 3. Experimental Setup

### Experimental Scenario

**Figure 4:**
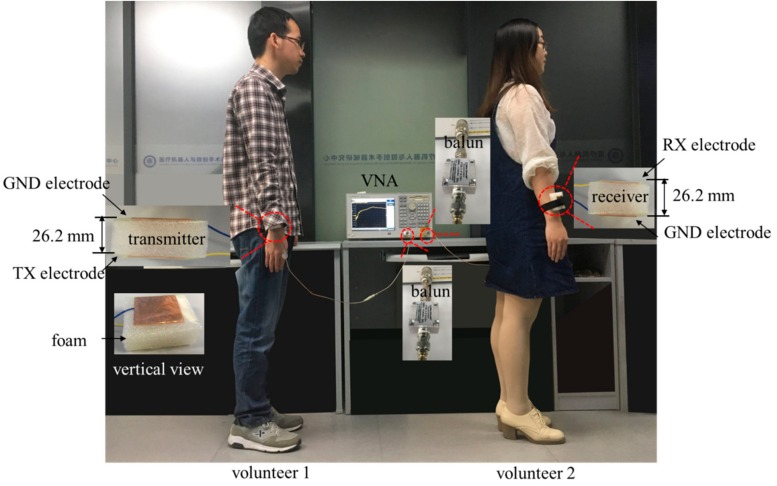
Experimental scenario.

To verify the validity of numerical simulations with an inhomogeneous human body model, experiment measurements were executed in this paper. Written informed consent was obtained from the volunteer before the measurement. As shown in Figure 4, two volunteers stood on the floor, and the distance between the volunteers was consistent with the simulation setup. The TX electrode, RX electrode, GND electrodes were fabricated by the copper. The distance between TX electrode/RX electrode and GND electrode was about 26.2 mm. The TX electrode of transmitter was attached on the forearm of volunteer 1 by the wristband, and the GND electrode of transmitter was exposed to the air. Furthermore, the transmitter was connected to the Port 1 of vector network analyzer (VNA, model number: E5061A, Agilent, CA, USA) by the cable and balun transformer with turn ratio of 1:1 (model number: FTB-1-1, Mini-Circuits, NY, USA). Similarly, the receiver was attached to volunteer 2 and connected to the Port 2 of VNA. The balun transformers were adopted in the experimental measurement for the reason that it could break the connection between the GND electrodes of transmitter and receiver [24,25], which corresponded to the simulation setup. The channel gain between transmitter and receiver was acquired by the VNA at four aforementioned scenarios in the frequency range 1 MHz to 90 MHz. Subsequently, the position of the transmitter or receiver was changed, and the channel gain was obtained at 44 MHz. Finally, the influence of the distance between electrode and human body was measured by the VNA at 44 MHz.

## 4. Results and Analysis

### 4.1. Propagation Characteristics of Different Scenarios

#### 4.1.1. The Channel Gain of Four Scenarios in the Frequency Range from 1 MHz to 90 MHz

**Figure 5:**
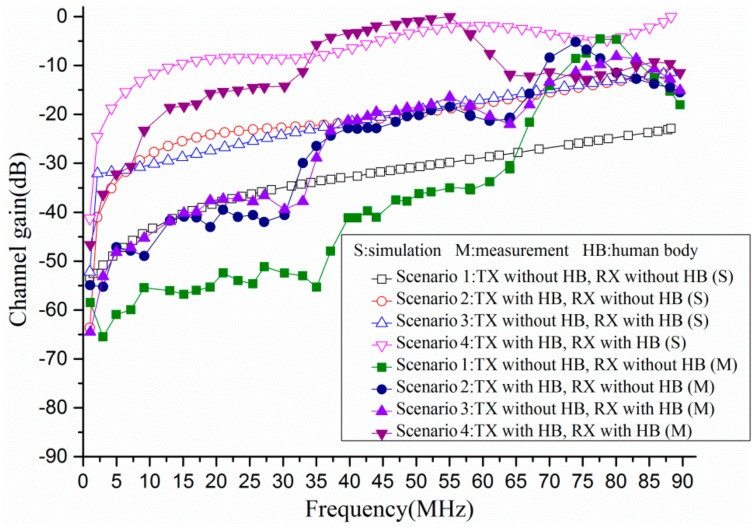
The propagation characteristics of four scenarios in the frequency range 1 MHz to 90 MHz.

Figure 5 illustrates the channel gain (in dB) of four scenarios through simulations and measurements when the frequency was from 1 MHz to 90 MHz. It is noteworthy that in order to better understand the propagation characteristics among four scenarios, the simulation results were pre-processed. Specifically, the maximum value of channel gain was set as a reference value, and then the difference between channel gain and reference value were obtained. Similarly, the measurement results were also pre-processed by the above method. According to the simulation results, the gain of all four scenarios had the smallest value at 1 MHz. When the frequency was below 65 MHz, as the frequency increased, the gain of all four scenarios increased. Additionally, it was interesting to observe that the gain of Scenario 2 and Scenario 3 was almost the same. On the other hand, as shown in Figure 5, the difference among the four scenarios was quite obvious when the frequency was below 65 MHz. The gain of Scenario 1 was the minimum, whereas the gain of Scenario 4 was the maximum. For instance, the gain of four scenarios was approximately −39.81 dB, −25.48 dB, −27.77 dB, −9.56 dB at 15 MHz, respectively. Furthermore, the gain was −30.85 dB, −19.60 dB, −18.51 dB, −2.99 dB when the frequency was 50 MHz. However, the channel gain of four scenarios was −24.53 dB, −13.41 dB, −12.95 dB, −4.21 dB individually at the frequency of 80 MHz. In addition, the results showed an agreement between the numerical simulations and experimental measurements for Scenario 2, Scenario 3 and Scenario 4. However, there was a little deviation for Scenario 1.

**Figure 6:**
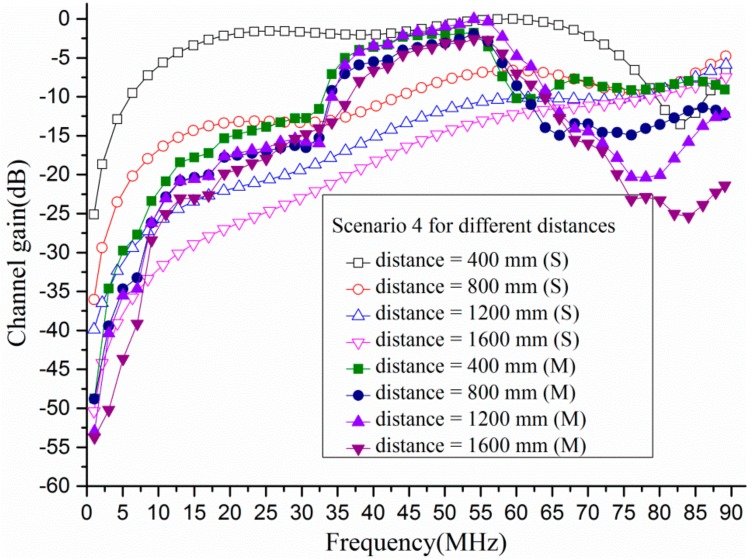
The propagation characteristics of different distance at Scenario 4.

Figure 6 shows the channel gain (in dB) of different distances (i.e., 400 mm, 800 mm, 1200 mm, 1600 mm) between transmitter and receiver at Scenario 4 when the frequency was from 1 MHz to 90 MHz. The simulation results and measurement results were also pre-processed by the aforementioned method. In terms of simulation results, it could be observed that as the distance between transmitter and receiver increased, the channel gain was decreased when the frequency was below 80 MHz. On the other hand, it was found that when the distance was beyond 800 mm, the difference of channel gain among different distances became small. For instance, the channel gain was more than −1.58 dB at 44 MHz when the distance was 400 mm. However, the channel gain was −9.77 dB, −13.39 dB, −16.01 dB respectively at 44 MHz when the distance was 800 mm, 1200 mm, 1600 mm. Thus, the distance between transmitter and receiver was taken as 800 mm in this paper.

#### 4.1.2. The Comparison of Four Scenarios at 44 MHz

To better understand the propagation characteristics when the human body was employed as an antenna, the input power, absorption power in human body, accepted power, input efficiency, accepted efficiency, and total efficiency of four scenarios were investigated when the frequency was at 44 MHz. The frequency of 44 MHz was selected as the research object for the following reasons. Firstly, according to [12,19,20], it was found that the human body resonated between 40 MHz and 60 MHz when it was exposed to the electromagnetic wave. Secondly, the height of the human body model in this paper was 1698 mm, which corresponded to the one-quarter wavelength when the frequency was 44 MHz. In this paper, the available power is the power which can be delivered by the voltage source given a conjugate impedance match between the simulation spaces. The input power is defined as the power delivered by a voltage source into the simulation space practically. It is worth noting that the available power was set as 1 W in the simulations. The input efficiency, accepted efficiency, and total efficiency are calculated as Equations (1)–(3), respectively. Table 2 lists the comparison of four scenarios.

(1) $$input\;efficiency=\frac{input\;power}{available\;power}\times 100\%$$

(2) $$accepted\;efficiency=\frac{accepted\;power}{input\;power}\times 100\%$$

(3) total efficiency=input efficiency×accepted efficiency

As listed in Table 2, it could be observed that the input efficiency of Scenario 1 was just 9.22 × 10^−4^%, which was 9.22 × 10^−6^ W when the TX electrode and RX electrode were placed in the air. The total efficiency of Scenario 1 was just 4.55 × 10^−8^%. Compared with Scenario 1, the input efficiency of Scenario 2 was increased to 7.53% when the TX electrode was attached to the human body. Therefore, it seems to indicate that more power could be injected into the simulation space when the human body was used as a transmitting antenna. Part of the power was absorbed by the human, and the other power was radiated out of the human body. The power was accepted by the RX electrode directly in Scenario 2, and the accepted efficiency was just 8.14 × 10^−6^%. Thus, the total efficiency of the Scenario was 6.13 × 10^−7^%. The input efficiency of Scenario 3 was 4.90 × 10^−5^%, which was similar to Scenario 1. However, the accepted efficiency was up to 1.24% when the RX electrode was attached to the human body. Thus, it was evident that the human body could harvest the power when it was used as a receiving antenna. The total efficiency of Scenario 3 was about 6.05 × 10^−7^%. For Scenario 4, the input efficiency was up to 7.53%. In addition, the accepted efficiency and total efficiency was 3.16 × 10^−4^% and 2.38 × 10^−5^%, respectively. Therefore, compared with other scenarios, the total efficiency of Scenario 4 was the greatest. To sum up, it is helpful to improve the input efficiency and accepted efficiency when the human body is used as an antenna.

#### 4.1.3. Electric Field Distribution of Different Distances at 44 MHz

**Figure 7:**
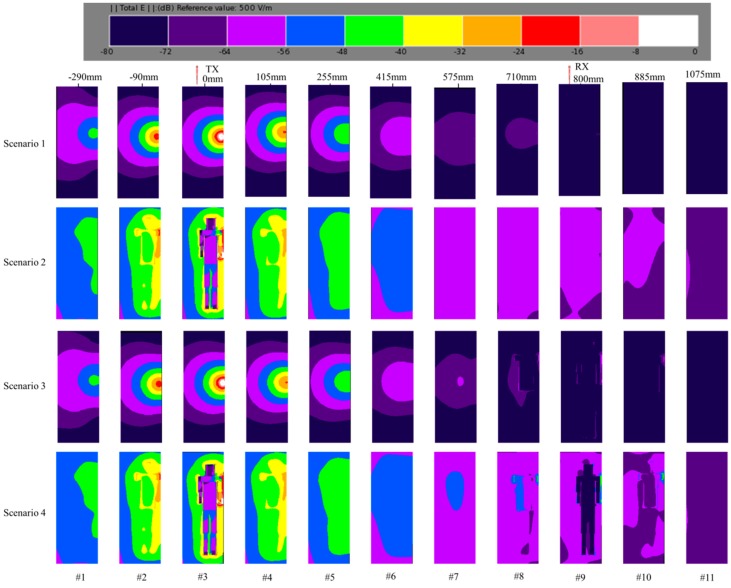
Electric field distribution of different distances at 44 MHz.

To intuitively analyze and compare the performances of four scenarios, the electric field distribution was studied in this section. Figure 7 demonstrates the electric field distribution of different distances for four scenarios when the frequency was 44 MHz. The original point (0 mm) was set as the position of transmitter. The distance from transmitter to receiver was 800 mm. The reference value of electric field intensity (EFI) was 500 V/m (i.e., 0 dB). As shown in the 3rd frame of Scenario 1, the EFI of TX electrode was approximately −20 dB, and the EFI near the TX electrode was from −32 dB to −64 dB. As the distance increased, the EFI decreased gradually. The EFI was from −72 dB to −76 dB when the distance was 800 mm. Moreover, the EFI declined to −80 dB at the distance of 1075 mm. Different from the electric field distribution in Scenario 1, the EFI of Scenario 2 in the 3rd frame was from −8 dB to −56 dB. Meanwhile, it could be observed that the EFI was almost the same when the distance was −90 mm and 105 mm. In addition, the EFI was quite similar when the distance was −290 mm and 25 mm. Thus, it was inferred that the power which is radiated out of the human body was omnidirectional. Compared with Scenario 1, the EFI was more than −56 dB when the distance was 800 mm, which indicated that the human body could be considered an effective transmitting antenna. On the other hand, it was interesting to observe that the electric field distribution of Scenario 3 was quite similar to Scenario 1 when the distance was below 415 mm, which was because both the TX electrodes of Scenario 1 and Scenario 3 were not attached to the human body. However, the EFI of Scenario 3 was greater than Scenario 1 when the distance was over 710 mm, which might be caused by the effect of the human body. As shown in the 8th, 9th and 10th frame of Scenario 3, the EFI around the human body was mainly from −56 dB to −80 dB. The electric field distribution of Scenario 4 was almost the same as Scenario 2 when the distance was below 415 mm. Because of the influence of the human body on the RX electrode, the EFI of Scenario 4 was greater than Scenario 2 when the distance was over 575 mm. The EFI of Scenario 4 was from −40 dB to −64 dB when the distance was 800 mm. Therefore, to sum up, the EFI is prominently enhanced when the TX electrode and RX electrode are attached to the human body.

### 4.2. Investigation on the Influence of Human Tissues

It is known that the human body consists of different tissues, and the dielectric properties (i.e., permittivity and conductivity) of different tissues are different. Thus, the influence of human tissue on propagation characteristics was studied in the paper. Considering that the principal tissues of the human body are skin, fat, muscle, bone, and organ [26,27], the aforementioned tissues were studied at 44 MHz in this paper. Specifically, the human body was modeled as a homogeneous model of skin, fat, muscle, bone, and organ, respectively. Furthermore, in terms of dielectric properties, there is no significant difference between the heart and other organs, such as liver, kidney, and so on [10]. Therefore, the dielectric property of the heart was used to represent other organs in this paper. The dielectric properties of different tissues at 44 MHz are listed in Table 3, and the total efficiency of different models are listed in Table 4.

As listed in Table 4 of Scenario 3, the total efficiency was associated with the conductivity and relative permittivity of model. For example, the total efficiency was 6.21 × 10^−7^%, 7.53 × 10^−7^% and 7.29 × 10^−7^% for skin model, muscle model and heart model. However, the total efficiency of fat model and bone model was relatively small, which was 3.96 × 10^−7^% and 3.79 × 10^−7^%. Furthermore, according to the dielectric properties of human tissues, the skin, muscle and heart are high-water-content tissues, whereas the fat and bone are low-water-content tissues [28,29]. Therefore, it was indicated that the high-water-content tissues played an important role in signal propagation when the human body was utilized as an antenna. In addition, as listed in in Table 4 of Scenario 4, the total efficiency was 2.27 × 10^−5^%, 3.80 × 10^−6^%,4.09 × 10^−5^%,3.85 × 10^−6^%,3.65 × 10^−5^%, when the human body was modeled as skin model, fat model, muscle model, bone model, and heart model, respectively. Thus, it also revealed the same conclusion that the high-water-content tissues, rather than the low-water-content tissues, played an important role in signal propagation.

### 4.3. Investigation on the Influence of Electrode Position

Table 5 lists the channel gain of Scenario 3 at 44 MHz when the TX electrode was placed in the air, and the RX electrode was attached on the different position of human body surface. The channel gain was pre-processed in this section so that we could study the influence of electrode position intuitively. Specifically, the value of channel gain was regarded as a reference value when the RX electrode was attached on the forearm. Subsequently, the differences between channel gain of different positions and reference value were obtained. As listed in Table 5, the channel gain was 0 dB, 1.285 dB, 2.521 dB, −0.871 dB, −0.871 dB, respectively in our simulations. Moreover, the difference between the minimum and maximum values was no more than 3.4 dB. Therefore, it could be inferred that the electrode position had little impact on channel gain when the human body was used as receiving antenna. In addition, the channel gain was 0 dB, −0.110 dB, −0.308 dB, −1.295 dB, −1.035 dB, respectively in the experimental measurements. The measurements also showed that the channel gain was almost independent of the electrode position.

Table 6 demonstrates the channel gain of Scenario 4 at 44 MHz when the TX electrode and RX electrode were placed on the different positions of human body surface. The channel gain was also pre-processed, and the value of channel gain was regarded as a reference value when the TX electrode and RX electrode were attached on the forearms of different humans. From the simulation results, it could be observed that the channel gain was 0 dB, −0.492 dB, −1.283 dB, −1.816 dB, −1.815 dB, −1.921 dB, and −1.921 dB at different positions. Therefore, it could be concluded that channel gain was also independent of electrode position when the human bodies were using as transmitting antenna and receiving antenna. Additionally, the channel gain was 0 dB, −0.510 dB, −0.999 dB, −1.700 dB, −2.296 dB, −3.412 dB, −3.390 dB in our measurements.

### 4.4. Investigation on the Influence of Distance between Electrode and Human Body

**Figure 8:**
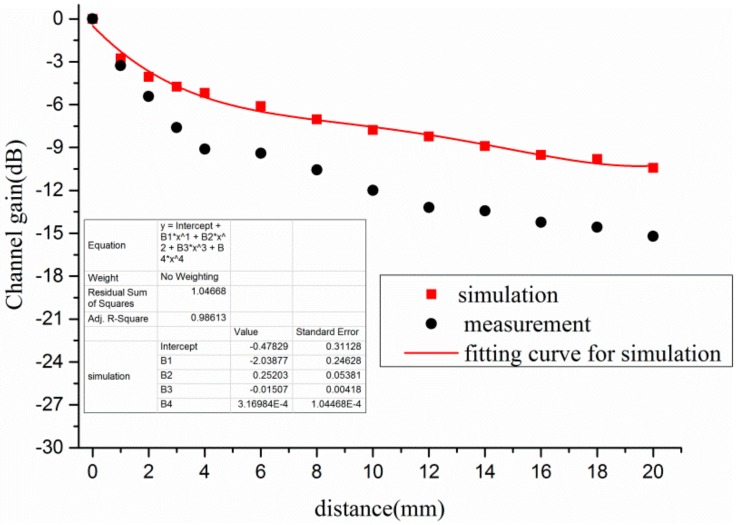
Influence of distance between electrode and human body at Scenario 3.

Figure 8 shows the results of channel gain as a function of the distance between RX electrode and human body for Scenario 3. In this section, the value of channel gain was regarded as a reference value when the distance was 0 mm. Subsequently, the differences between channel gain of different distances and reference value were obtained. In terms of the simulation results, the channel gain was 0 dB when the RX electrode was closely attached on the human body. As the distance increased, the channel gain decreased. The channel gain was declined from 0 dB to −7.77 dB at the distance of 10 mm. Subsequently, the decrease of channel gain became slow while the distance was over 10 mm. The channel gain was −8.22 dB, −8.89 dB, −9.52 dB, −9.81 dB, and −10.43 dB respectively at the distance of 12 mm, 14 mm, 16 mm, 18 mm, and 20 mm. According to the correlation between channel gain and distance, the correlation could be fitted by the quartic polynomial, as shown in Equation (4). In addition, the measurement result also showed the same decreasing trend.

(4) $$y=-0.478-2.039\cdot x+0.252\cdot x^{2}-0.015\cdot x^{3}+3.169\times 10^{-4}\cdot x^{4}$$

where *y* is the channel gain, and *x* is the distance between RX electrode and human body.

**Figure 9:**
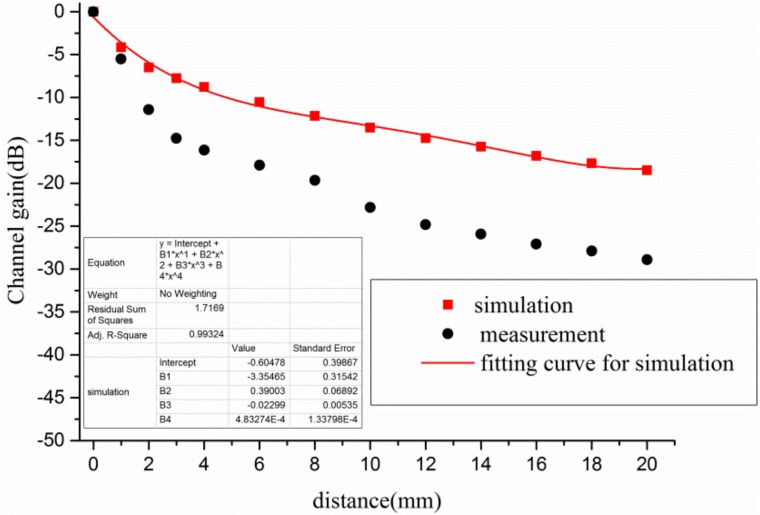
Influence of distance between electrode and human body at Scenario 4.

The channel gain of different distances between electrode and human body at Scenario 4 is plotted in Figure 9. As shown in Figure 9, the changing trend of channel gain was almost the same between simulation and measurement. According to the simulation, the channel gain was 0 dB when both the TX electrode and RX electrode were closely attached on the human bodies. However, the channel gain decreased sharply as the distance increased. For instance, the channel gain declined from 0 dB to −8.78 dB when the distance was 4 mm. Furthermore, the channel gain was −13.52 dB and −18.49 dB when the distance was 10 mm and 20 mm individually. Thus, it was revealed that the distance had a great impact on the channel gain when the human bodies were used as transmitting antenna and receiving antenna. To better evaluate the influence of different distances on channel gain, the relationship between channel gain and the distance was fitted by the polynomial, as shown in Equation (5).

(5) $$y=-0.605-3.355\cdot x+0.390\cdot x^{2}-0.023\cdot x^{3}+4.833\times 10^{-4}\cdot x^{4}$$

where *y* is the channel gain, and *x* is the distance between electrode and human body.

### 4.5. Discussion

The channel gain shown in Figure 5 revealed that the channel gain of different scenarios varied with the frequency. Furthermore, due to the influence of the human body, the channel gain of Scenario 2, Scenario 3, and Scenario 4 was much greater than the channel gain of Scenario 1 when the frequency was below 90 MHz. Thus, it was indicated that the human body could be used as a transmitting antenna or receiving antenna to achieve the signal transmission, which was helpful to improve the channel gain. However, the difference of channel gain among the four scenarios became small when the frequency was beyond 65 MHz, which might be explained by the relationship of power propagation and absorption. As the frequency increased, the power was more easily transmitted through air. Moreover, there was no power absorption by the human body in Scenario 1. Thus, the channel gain of Scenario 1 increased sharply. On the other hand, the power was absorbed by the human body in Scenario 2, Scenario 3 and Scenario 4. Therefore, the difference of the four scenarios became small when the frequency was beyond 65 MHz.

On the other hand, as demonstrated in Figure 5, the channel gain of Scenario 2 and Scenario 3 was almost the same in the frequency range 1 MHz to 90 MHz, which might be explained by the reciprocity theorem of antenna [30,31]. According to the reciprocity theorem, the characteristics remain the same when an antenna is used for transmitting signal or receiving signal under the conduction that the antenna is not nonlinear material. Due to the fact that the human tissues are linear material, the propagation characteristics were similar when the human body was used as a transmitting antenna in Scenario 2 and used as a receiving antenna in Scenario 3.

## 5. Conclusions

This paper presented the propagation characteristics of four scenarios through numerical simulations with an inhomogeneous model and in-situ experiments. Compared with the channel gain of Scenario 1 in which both the TX electrode and RX electrode were placed in the air, the results showed that the channel gain was greater when the TX electrode or RX electrode was attached to the human body in the frequency range 1 MHz to 90 MHz. Therefore, it could be inferred that the human body could be regarded as a transmitting antenna or receiving antenna to achieve the signal transmission. Specifically, the input efficiency of Scenario 2 was up to 7.53% at 44 MHz when the human body was used as a transmitting antenna, whereas the input efficiency of Scenario 1 was just 9.22 × 10^−4^% without the human body. Similarly, the accepted efficiency of Scenario 3 rose from 4.90 × 10^−3^% to 1.24% when the human body was utilized as a receiving antenna. Furthermore, in terms of the electric field distribution, it could be observed that the electric field intensity around the human body was enhanced dramatically when TX electrode or RX electrode was attached on the human body, which also revealed the feasibility of using the human body as a transmitting antenna or receiving antenna. In addition, the factors affecting propagation characteristics were explored when the human body was used as an antenna in this paper. The results indicated that the high-water-content tissues, such as skin, muscle, heart, and so on, played an important role in signal propagation, which was helpful to improve the channel gain. The channel gain of Scenario 3 was 0 dB, 1.285 dB, 2.521 dB, −0.871 dB, and −0.871 dB, respectively when the RX electrode was attached to different position of the human body. In addition, the channel gain of Scenario 4 was 0 dB, −0.492 dB, −1.283 dB, −1.816 dB, −1.815 dB, −1.921 dB, and −1.921 dB at different electrode positions. Therefore, it seemed to indicate that the channel gain was insensitive with electrode position when the human body was employed as an antenna. However, the channel gain was associated with the distance between electrode and human body. As the distance increased, the channel gain decreased rapidly, and the relationship could be represented by a quartic polynomial. In summary, the human body can be used as an antenna to achieve wireless transmission, which may provide a promising communication solution in BSN with the advantages of low cost and convenience. In the near future, we will investigate the influence of the dynamic human body when it is used as an antenna to achieve wireless transmission.

## Figures and Tables

**Table 1:** The tissue layers and thicknesses of different body parts in human body model (Unit: mm).

Body Parts	Skin	Fat	Muscle	Bone	Grey Matter	Heart
head	4	-	9.5	20.5	51	-
chest	2	4	38	46	-	-
abdomen	2	7	25	-	-	56
upper arm	2	6.1	20.3	9.1	-	-
forearm	2	4.3	14.9	6.3	-	-
thigh	2	8.8	28.7	13	-	-
lower leg	2	6.3	20.8	9.4	-	-

**Table 2:** Comparison of four scenarios at 44 MHz.

	Scenario 1	Scenario 2	Scenario 3	Scenario 4
input power(W)	9.22 × 10^−6^	0.0753	4.90 × 10^−7^	0.07529
total absorption power (W)	-	0.05989	3.312 × 10^−7^	0.05991
TX absorption power (W)	-	0.05989	-	0.05989
RX absorption power (W)	-	-	3.312 × 10^−7^	0.00002
accepted power (W)	4.55 × 10^−10^	6.13 × 10^−9^	6.06 × 10^−9^	2.378 × 10^−7^
input efficiency	9.22 × 10^−4^%	7.53%	4.90 × 10^−5^%	7.53%
accepted efficiency	4.90 × 10^−3^%	8.14 × 10^−6^%	1.24%	3.16 × 10^−4^%
total efficiency	4.55 × 10^−8^%	6.13 × 10^−7^%	6.05 × 10^−7^%	2.38 × 10^−5^%

**Table 3:** The dielectric properties of different tissues at 44 MHz.

Biological Tissue	Skin	Fat	Muscle	Bone	Heart
relative permittivity	116.54	7.1176	80.069	18.4	124.85
conductivity (S/m)	0.38954	0.034347	0.67297	0.055928	0.63689

**Table 4:** Total efficiency of different human body models using as an antenna at 44 MHz.

Total Efficiency	Skin Model	Fat Model	Muscle Model	Bone Model	Heart Model	Inhomogeneous Model
Scenario 3	6.21 × 10^−7^%	3.96 × 10^−7^%	7.53 × 10^−7^%	3.79 × 10^−7^%	7.29 × 10^−7^%	6.05 × 10^−7^%
Scenario 4	2.27 × 10^−5^%	3.80 × 10^−6^%	4.09 × 10^−5^%	3.85 × 10^−6^%	3.65 × 10^−5^%	2.38 × 10^−5^%

**Table 5:** The influence of electrode position at 44 MHz for Scenario 3.

TX Electrode	Air	Air	Air	Air	Air
RX electrode	forearm	upper arm	thigh	abdomen	back
simulation (dB)	0	1.285	2.521	−0.871	−0.871
measurement(dB)	0	−0.110	−0.308	−1.295	−1.035

**Table 6:** The influence of electrode position at 44 MHz for Scenario 4.

TX Electrode	Forearm	Forearm	Forearm	Forearm	Forearm	Abdomen	Back
RX electrode	forearm	upper arm	thigh	abdomen	back	abdomen	back
simulation (dB)	0	−0.492	−1.283	−1.816	−1.815	−1.921	−1.921
measurement(dB)	0	−0.510	−0.999	−1.700	−2.296	−3.412	−3.390
